# Tris(*N*-benzoyl-*N*′,*N*′-diphenyl­thio­ureato-κ^2^ *O*,*S*)cobalt(III)

**DOI:** 10.1107/S160053680800531X

**Published:** 2008-02-29

**Authors:** Hiram Pérez, Yvonne Mascarenhas, Ana María Plutín, Rodrigo de Souza Corrêa, Julio Duque

**Affiliations:** a Departamento de Química Inorgánica, Facultad de Química, Universidad de la Habana, Habana 10400, Cuba; b Instituto de Física de São Carlos, Universidade de São Paulo, São Carlos, Brazil; c Laboratorio de Síntesis Orgánica, Facultad de Química, Universidad de la Habana, Habana 10400, Cuba; d Instituto de Ciencia y Tecnología de Materiales, Universidad de la Habana, Habana 10400, Cuba

## Abstract

In the title compound, [Co(C_20_H_15_N_2_OS)_3_], the Co^III^ atom is coordinated by the S and O atoms of three *N*-benzoyl-*N*′,*N*′-diphenyl­thio­urea ligands in a slightly distorted octa­hedral geometry. The O and S atoms are in *cis* positions, while the positions between the O and S atoms are *trans*.

## Related literature

For general background and related structures, see: Arslan *et al.* (2003 ▶); Jia *et al.* (2007 ▶). For ligand synthesis, see: Hernández *et al.* (2003 ▶).
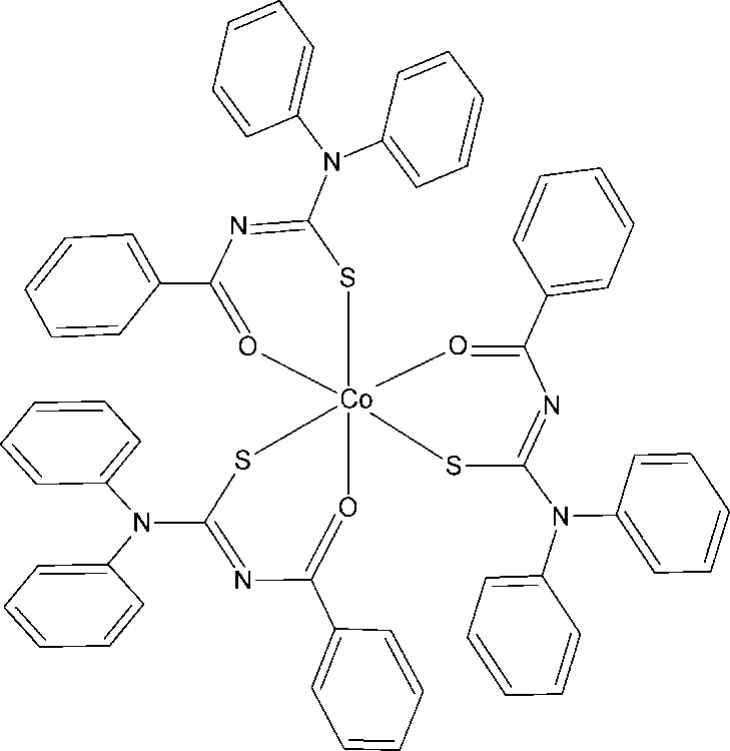


## Experimental

### 

#### Crystal data

[Co(C_20_H_15_N_2_OS)_3_]
*M* *_r_* = 1053.13
Triclinic, 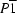

*a* = 10.460 (1) Å
*b* = 13.591 (5) Å
*c* = 20.515 (5) Å
α = 93.371 (2)°
β = 97.652 (5)°
γ = 112.212 (5)°
*V* = 2657.2 (12) Å^3^
*Z* = 2
Mo *K*α radiation
μ = 0.49 mm^−1^
*T* = 150 (2) K
0.22 × 0.12 × 0.03 mm

#### Data collection

Nonius KappaCCD diffractometer
Absorption correction: Gaussian (Coppens *et al.*, 1965 ▶) *T*_min_ = 0.862, *T*_max_ = 0.971
16799 measured reflections
9325 independent reflections
7633 reflections with *I* > 2σ(*I*)
*R*_int_ = 0.057

#### Refinement

*R*[*F* ^2^ > 2σ(*F* ^2^)] = 0.057
*wR*(*F* ^2^) = 0.162
*S* = 1.13
9325 reflections
654 parameters
H-atom parameters constrained
Δρ_max_ = 0.34 e Å^−3^
Δρ_min_ = −0.6 e Å^−3^

### 

Data collection: *COLLECT* (Enraf–Nonius, 2000); cell refinement: *DENZO* (Otwinowski & Minor, 1997 ▶); data reduction: *DENZO*; program(s) used to solve structure: *SHELXS97* (Sheldrick, 2008 ▶); program(s) used to refine structure: *SHELXL97* (Sheldrick, 2008 ▶); molecular graphics: *ORTEP-3 for Windows* (Farrugia, 1997 ▶); software used to prepare material for publication: *WinGX* (Farrugia, 1999 ▶).

## Supplementary Material

Crystal structure: contains datablocks global, I. DOI: 10.1107/S160053680800531X/hy2119sup1.cif

Structure factors: contains datablocks I. DOI: 10.1107/S160053680800531X/hy2119Isup2.hkl

Additional supplementary materials:  crystallographic information; 3D view; checkCIF report

## Figures and Tables

**Table:** 

O1—Co1	1.920 (2)
O2—Co1	1.923 (2)
O3—Co1	1.934 (2)
S1—Co1	2.2153 (9)
S2—Co1	2.2169 (11)
S3—Co1	2.1985 (10)

**Table:** 

O1—Co1—O2	85.41 (9)
O1—Co1—O3	87.12 (9)
O2—Co1—O3	85.99 (9)
O1—Co1—S3	89.85 (7)
O2—Co1—S3	175.21 (7)
O3—Co1—S3	93.07 (7)
O1—Co1—S1	95.85 (7)
O2—Co1—S1	92.24 (7)
O3—Co1—S1	176.42 (7)
S3—Co1—S1	88.94 (4)
O1—Co1—S2	177.27 (7)
O2—Co1—S2	92.85 (7)
O3—Co1—S2	90.66 (7)
S3—Co1—S2	91.86 (4)
S1—Co1—S2	86.31 (4)

**Fig. 1.**
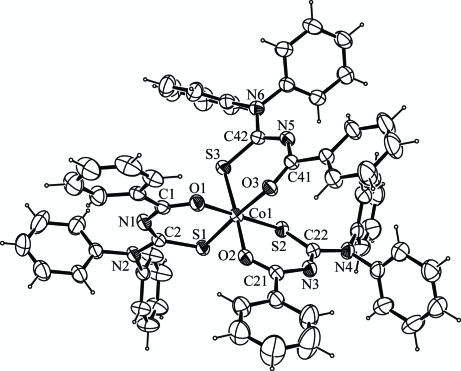
The molecular structure of the title compound. Displacement ellipsoids are drawn at the 50% probability level.

**Fig. 2.**
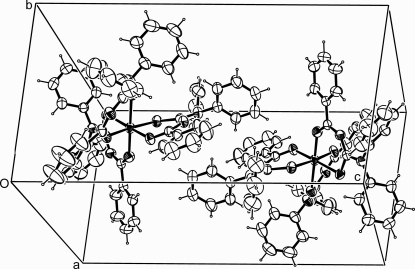
View of the unit cell of the title compound.

## supplementary crystallographic information

### Comment

Substituted *N*-acylthioureas are well known as chelating agents. Over recent years, many transition metal complexes with thiourea derivatives have been reported (Arslan *et al.*, 2003), because this kind of ligands display a remarkably rich coordination chemistry.

In this paper, we report the crystal structure of the title compound (Fig. 1), which presents an octahedral environment about the Co^III^ atom with the ligands coordinating in a relatively distorted manner (Table 1). The Co—S bond lengths lie within the range of those found in the related structure (Jia *et al.*, 2007). The lengths of C—O, C—S and C—N bonds in the chelate rings are between characteristic single and double bond lengths; they are shorter than single bond and longer than double bond. These results can be explained by the existence of delocalization in the chelate rings. Fig. 2 shows the arrangement of the complex molecules in the unit cell.

### Experimental

*N*-benzoyl-*N*',*N*'-diphenylthiourea ligand was synthesized according to a procedure described by Hernández *et al.* (2003), by converting benzoyl chloride into benzoyl isothiocyanate and then condensing with an appropriate amine. To an ethanol solution (30 ml) containing the ligand (0.66 g, 2 mmol) was added an ethanol solution of Co(CH_3_COO)_2_.4H_2_O (0.25 g, 1 mmol). The solution was stirred at room temperature for 2 h, and at once a solution of NaOH (1 N) was added to adjust pH to the neutral value. The mixture was filtered and the filtrate was evaporated under reduced pressure to give a green solid, which was washed with acetone. Single crystals were obtained by slow evaporation of a chloroform/*N*,*N*-diphenylformamide solution (1:1, *v*/*v*) of the complex.

### Refinement

H atoms were positioned geometrically and refined as riding atoms, with C—H = 0.93 Å and *U*_iso_(H) = 1.2*U*_eq_(C).

### Crystal data

**Table:**

[Co(C_20_H_15_N_2_OS)_3_]	*Z* = 2
*M**_r_* = 1053.13	*F*_000_ = 1092
Triclinic, *P*1	*D*_x_ = 1.316 Mg m^−^^3^
Hall symbol: -P 1	Mo *K*α radiation λ = 0.71073 Å
*a* = 10.460 (1) Å	Cell parameters from 288 reflections
*b* = 13.591 (5) Å	θ = 2.9–26.4º
*c* = 20.515 (5) Å	µ = 0.49 mm^−^^1^
α = 93.371 (2)º	*T* = 150 (2) K
β = 97.652 (5)º	Block, green
γ = 112.212 (5)º	0.22 × 0.12 × 0.03 mm
*V* = 2657.2 (12) Å^3^	

### Data collection

**Table:**

Nonius KappaCCD diffractometer	7633 reflections with *I* > 2σ(*I*)
Radiation source: fine-focus sealed tube	*R*_int_ = 0.057
Monochromator: graphite	θ_max_ = 25º
φ and ω scans	θ_min_ = 3.0º
Absorption correction: Gaussian(Coppens *et al.*, 1965)	*h* = −12→12
*T*_min_ = 0.862, *T*_max_ = 0.971	*k* = −16→16
16799 measured reflections	*l* = −24→24
9325 independent reflections	

### Refinement

**Table:**

Refinement on *F*^2^	H-atom parameters constrained
Least-squares matrix: full	*w* = 1/[σ^2^(*F*_o_^2^) + (0.0786*P*)^2^ + 0.1153*P*] where *P* = (*F*_o_^2^ + 2*F*_c_^2^)/3
*R*[*F*^2^ > 2σ(*F*^2^)] = 0.057	(Δ/σ)_max_ < 0.001
*wR*(*F*^2^) = 0.162	Δρ_max_ = 0.34 e Å^−^^3^
*S* = 1.13	Δρ_min_ = −0.6 e Å^−^^3^
9325 reflections	Extinction correction: none
654 parameters	

### Special details

**Table:**

Geometry. All e.s.d.'s (except the e.s.d. in the dihedral angle between two l.s. planes) are estimated using the full covariance matrix. The cell e.s.d.'s are taken into account individually in the estimation of e.s.d.'s in distances, angles and torsion angles; correlations between e.s.d.'s in cell parameters are only used when they are defined by crystal symmetry. An approximate (isotropic) treatment of cell e.s.d.'s is used for estimating e.s.d.'s involving l.s. planes.

### Fractional atomic coordinates and isotropic or equivalent isotropic displacement parameters (Å^2^)

**Table:**

	*x*	*y*	*z*	*U*_iso_*/*U*_eq_	
C1	0.6058 (3)	0.4720 (2)	0.18641 (13)	0.0483 (6)	
C2	0.3964 (3)	0.4260 (2)	0.10944 (13)	0.0469 (6)	
C3	0.7327 (3)	0.4463 (2)	0.19797 (13)	0.0522 (7)	
C4	0.8459 (3)	0.5073 (3)	0.24551 (16)	0.0691 (9)	
H4	0.8422	0.5638	0.272	0.083*	
C5	0.9652 (4)	0.4848 (4)	0.2541 (2)	0.0955 (14)	
H5	1.0417	0.5265	0.2861	0.115*	
C6	0.9707 (5)	0.4024 (5)	0.2161 (2)	0.1075 (16)	
H6	1.0511	0.3878	0.2221	0.129*	
C7	0.8597 (5)	0.3404 (4)	0.1690 (2)	0.1022 (15)	
H7	0.8646	0.2838	0.1432	0.123*	
C8	0.7403 (4)	0.3618 (3)	0.15960 (18)	0.0731 (9)	
H8	0.6646	0.3194	0.1275	0.088*	
C9	0.3767 (3)	0.2802 (3)	0.02958 (14)	0.0563 (7)	
C10	0.3225 (5)	0.1780 (3)	0.0435 (2)	0.0945 (13)	
H10	0.2516	0.1568	0.0689	0.113*	
C11	0.3739 (7)	0.1046 (4)	0.0193 (3)	0.134 (2)	
H11	0.3365	0.0339	0.0282	0.161*	
C12	0.4794 (7)	0.1369 (5)	−0.0175 (3)	0.1169 (17)	
H12	0.5139	0.0881	−0.0334	0.14*	
C13	0.5332 (5)	0.2388 (4)	−0.0307 (2)	0.0900 (12)	
H13	0.6056	0.2606	−0.0553	0.108*	
C14	0.4812 (3)	0.3111 (3)	−0.00779 (16)	0.0678 (8)	
H14	0.5173	0.3812	−0.0178	0.081*	
C15	0.1953 (3)	0.3554 (2)	0.01770 (14)	0.0535 (7)	
C16	0.1959 (4)	0.3985 (3)	−0.04118 (17)	0.0737 (9)	
H16	0.2796	0.4305	−0.0569	0.088*	
C17	0.0722 (4)	0.3942 (4)	−0.07690 (19)	0.0913 (12)	
H17	0.0735	0.4242	−0.1165	0.11*	
C18	−0.0503 (4)	0.3472 (4)	−0.0554 (2)	0.0982 (14)	
H18	−0.1333	0.3441	−0.0801	0.118*	
C19	−0.0511 (4)	0.3043 (5)	0.0032 (2)	0.1144 (18)	
H19	−0.1353	0.272	0.0186	0.137*	
C20	0.0719 (4)	0.3084 (4)	0.03974 (18)	0.0858 (12)	
H20	0.0703	0.279	0.0796	0.103*	
C21	0.6062 (3)	0.8182 (2)	0.19445 (14)	0.0567 (7)	
C22	0.4123 (3)	0.8152 (2)	0.24214 (14)	0.0553 (7)	
C23	0.7319 (3)	0.8900 (3)	0.16920 (16)	0.0664 (8)	
C24	0.8101 (4)	0.8470 (3)	0.13729 (18)	0.0740 (9)	
H24	0.7836	0.7731	0.1319	0.089*	
C25	0.9257 (4)	0.9102 (4)	0.1135 (2)	0.1016 (13)	
H25	0.9754	0.8797	0.0908	0.122*	
C26	0.9668 (6)	1.0168 (5)	0.1230 (4)	0.153 (2)	
H26	1.0469	1.0604	0.1079	0.184*	
C27	0.8913 (6)	1.0628 (4)	0.1553 (4)	0.159 (3)	
H27	0.9202	1.1368	0.1616	0.19*	
C28	0.7733 (5)	0.9983 (3)	0.1780 (3)	0.1106 (14)	
H28	0.7217	1.0287	0.1994	0.133*	
C29	0.4254 (4)	0.9955 (3)	0.27415 (18)	0.0668 (9)	
C30	0.4007 (5)	1.0494 (3)	0.2228 (2)	0.1031 (14)	
H30	0.3414	1.012	0.1838	0.124*	
C31	0.4642 (6)	1.1594 (4)	0.2294 (3)	0.1232 (18)	
H31	0.4484	1.196	0.1943	0.148*	
C32	0.5481 (6)	1.2144 (4)	0.2854 (3)	0.1056 (15)	
H32	0.5874	1.2888	0.2896	0.127*	
C33	0.5761 (5)	1.1622 (3)	0.3359 (2)	0.0985 (13)	
H33	0.6372	1.2006	0.3742	0.118*	
C34	0.5135 (4)	1.0506 (3)	0.3307 (2)	0.0827 (10)	
H34	0.532	1.0144	0.3655	0.099*	
C35	0.2295 (4)	0.8392 (2)	0.29742 (17)	0.0644 (8)	
C36	0.2357 (4)	0.8093 (3)	0.36055 (18)	0.0777 (10)	
H36	0.32	0.8131	0.3842	0.093*	
C37	0.1148 (5)	0.7735 (3)	0.3880 (2)	0.0885 (12)	
H37	0.1181	0.752	0.4302	0.106*	
C38	−0.0086 (5)	0.7691 (3)	0.3545 (3)	0.0952 (13)	
H38	−0.0887	0.7461	0.3739	0.114*	
C39	−0.0140 (5)	0.7985 (4)	0.2924 (3)	0.1089 (15)	
H39	−0.0985	0.795	0.2691	0.131*	
C40	0.1056 (4)	0.8338 (3)	0.2636 (2)	0.0895 (12)	
H40	0.1011	0.8537	0.221	0.107*	
C41	0.5351 (3)	0.6913 (2)	0.36616 (13)	0.0522 (7)	
C42	0.3583 (3)	0.5191 (2)	0.35847 (13)	0.0518 (7)	
C43	0.6139 (3)	0.7917 (2)	0.41223 (14)	0.0572 (7)	
C44	0.7273 (4)	0.8716 (3)	0.39512 (18)	0.0734 (9)	
H44	0.7539	0.8634	0.3543	0.088*	
C45	0.8020 (5)	0.9639 (3)	0.4379 (2)	0.1027 (15)	
H45	0.879	1.0174	0.4262	0.123*	
C46	0.7623 (6)	0.9761 (4)	0.4975 (2)	0.1163 (17)	
H46	0.8138	1.0375	0.5269	0.14*	
C47	0.6478 (6)	0.8992 (4)	0.5144 (2)	0.1141 (17)	
H47	0.6191	0.9098	0.5543	0.137*	
C48	0.5748 (4)	0.8064 (3)	0.47290 (17)	0.0822 (11)	
H48	0.4987	0.753	0.4854	0.099*	
C49	0.2637 (3)	0.4864 (2)	0.46147 (15)	0.0583 (7)	
C50	0.1969 (4)	0.5550 (3)	0.46646 (17)	0.0748 (9)	
H50	0.1662	0.5801	0.4288	0.09*	
C51	0.1750 (4)	0.5869 (3)	0.52809 (19)	0.0847 (11)	
H51	0.1293	0.6333	0.5318	0.102*	
C52	0.2211 (4)	0.5497 (3)	0.58357 (19)	0.0822 (11)	
H52	0.2062	0.5708	0.6249	0.099*	
C53	0.2890 (4)	0.4818 (3)	0.57807 (18)	0.0847 (11)	
H53	0.3209	0.4573	0.6158	0.102*	
C54	0.3106 (4)	0.4492 (3)	0.51650 (17)	0.0751 (9)	
H54	0.3563	0.4028	0.5127	0.09*	
C55	0.2017 (4)	0.3380 (3)	0.37298 (16)	0.0625 (8)	
C56	0.0584 (4)	0.2992 (3)	0.3611 (2)	0.0851 (11)	
H56	0.0121	0.3441	0.3689	0.102*	
C57	−0.0175 (5)	0.1915 (4)	0.3370 (3)	0.1113 (16)	
H57	−0.1148	0.1646	0.3282	0.134*	
C58	0.0513 (7)	0.1254 (4)	0.3263 (3)	0.1168 (17)	
H58	0.0004	0.0536	0.3105	0.14*	
C59	0.1918 (6)	0.1639 (4)	0.3386 (3)	0.1068 (15)	
H59	0.2378	0.1187	0.3311	0.128*	
C60	0.2684 (4)	0.2704 (3)	0.3622 (2)	0.0830 (11)	
H60	0.3657	0.2964	0.3708	0.1*	
N1	0.5092 (2)	0.4108 (2)	0.13594 (11)	0.0542 (6)	
N2	0.3234 (2)	0.3561 (2)	0.05438 (11)	0.0532 (6)	
N3	0.5350 (3)	0.8671 (2)	0.22344 (13)	0.0613 (6)	
N4	0.3564 (3)	0.8799 (2)	0.26916 (13)	0.0634 (7)	
N5	0.4330 (3)	0.61702 (19)	0.38961 (11)	0.0557 (6)	
N6	0.2803 (3)	0.4495 (2)	0.39679 (12)	0.0597 (6)	
O1	0.6057 (2)	0.54725 (16)	0.22486 (10)	0.0576 (5)	
O2	0.5835 (2)	0.72042 (17)	0.18653 (10)	0.0611 (5)	
O3	0.5765 (2)	0.68743 (16)	0.31129 (9)	0.0561 (5)	
S1	0.33442 (8)	0.52013 (7)	0.13271 (4)	0.0583 (2)	
S2	0.31150 (8)	0.68008 (6)	0.23515 (4)	0.0580 (2)	
S3	0.34463 (8)	0.46920 (6)	0.27777 (4)	0.0569 (2)	
Co1	0.46544 (4)	0.60548 (3)	0.228631 (17)	0.05040 (14)	

### Atomic displacement parameters (Å^2^)

**Table:**

	*U*^11^	*U*^22^	*U*^33^	*U*^12^	*U*^13^	*U*^23^
C1	0.0521 (15)	0.0543 (16)	0.0415 (14)	0.0212 (13)	0.0147 (12)	0.0109 (12)
C2	0.0520 (15)	0.0505 (15)	0.0406 (14)	0.0206 (12)	0.0138 (12)	0.0066 (11)
C3	0.0510 (15)	0.0656 (18)	0.0443 (15)	0.0248 (14)	0.0137 (12)	0.0123 (13)
C4	0.0580 (18)	0.095 (3)	0.0499 (17)	0.0253 (17)	0.0081 (14)	0.0105 (16)
C5	0.055 (2)	0.162 (4)	0.068 (2)	0.041 (2)	0.0051 (17)	0.021 (3)
C6	0.082 (3)	0.190 (5)	0.086 (3)	0.088 (3)	0.021 (2)	0.023 (3)
C7	0.096 (3)	0.153 (4)	0.094 (3)	0.089 (3)	0.018 (3)	0.008 (3)
C8	0.069 (2)	0.089 (3)	0.071 (2)	0.0436 (19)	0.0102 (17)	0.0014 (18)
C9	0.0630 (17)	0.0615 (18)	0.0483 (16)	0.0301 (15)	0.0080 (13)	−0.0010 (13)
C10	0.122 (3)	0.076 (3)	0.107 (3)	0.053 (2)	0.046 (3)	0.026 (2)
C11	0.185 (6)	0.085 (3)	0.172 (6)	0.084 (4)	0.059 (5)	0.036 (3)
C12	0.165 (5)	0.130 (4)	0.109 (4)	0.112 (4)	0.037 (4)	0.008 (3)
C13	0.099 (3)	0.129 (4)	0.068 (2)	0.074 (3)	0.015 (2)	−0.001 (2)
C14	0.070 (2)	0.084 (2)	0.0570 (18)	0.0390 (18)	0.0137 (15)	−0.0014 (16)
C15	0.0559 (16)	0.0611 (18)	0.0451 (15)	0.0265 (14)	0.0050 (12)	0.0002 (13)
C16	0.071 (2)	0.093 (3)	0.0577 (19)	0.0288 (19)	0.0153 (16)	0.0235 (18)
C17	0.088 (3)	0.127 (4)	0.066 (2)	0.048 (3)	0.007 (2)	0.038 (2)
C18	0.074 (2)	0.158 (4)	0.074 (3)	0.059 (3)	0.004 (2)	0.028 (3)
C19	0.061 (2)	0.193 (5)	0.090 (3)	0.044 (3)	0.018 (2)	0.050 (3)
C20	0.067 (2)	0.133 (4)	0.061 (2)	0.037 (2)	0.0155 (17)	0.036 (2)
C21	0.0709 (19)	0.0569 (18)	0.0443 (15)	0.0253 (15)	0.0160 (14)	0.0032 (13)
C22	0.0697 (18)	0.0557 (17)	0.0484 (16)	0.0313 (15)	0.0164 (14)	0.0054 (13)
C23	0.073 (2)	0.063 (2)	0.0609 (19)	0.0215 (16)	0.0237 (16)	0.0033 (15)
C24	0.071 (2)	0.078 (2)	0.072 (2)	0.0268 (18)	0.0225 (18)	0.0010 (18)
C25	0.088	0.097 (3)	0.125 (4)	0.031 (2)	0.053 (2)	0.011 (3)
C26	0.133	0.103 (4)	0.232 (7)	0.025 (3)	0.120 (4)	0.034 (4)
C27	0.137	0.071 (3)	0.278 (9)	0.022 (3)	0.121 (4)	0.029 (4)
C28	0.12	0.065 (2)	0.153 (4)	0.026 (2)	0.073 (3)	0.013 (3)
C29	0.081 (2)	0.0542 (18)	0.077 (2)	0.0317 (17)	0.0355 (18)	0.0091 (16)
C30	0.143 (4)	0.074 (3)	0.085 (3)	0.034 (3)	0.016 (3)	0.022 (2)
C31	0.173 (5)	0.069 (3)	0.122 (4)	0.036 (3)	0.026 (4)	0.042 (3)
C32	0.135 (4)	0.059 (2)	0.117 (4)	0.022 (3)	0.047 (3)	0.017 (3)
C33	0.102 (3)	0.075 (3)	0.097 (3)	0.013 (2)	0.019 (2)	−0.005 (2)
C34	0.096 (3)	0.064 (2)	0.084 (3)	0.026 (2)	0.019 (2)	0.0080 (19)
C35	0.081 (2)	0.0531 (18)	0.071 (2)	0.0345 (16)	0.0291 (17)	0.0042 (15)
C36	0.093 (3)	0.080 (2)	0.069 (2)	0.038 (2)	0.0292 (19)	0.0081 (18)
C37	0.126 (3)	0.078 (3)	0.077 (2)	0.045 (2)	0.051 (3)	0.0134 (19)
C38	0.105 (3)	0.080 (3)	0.126 (4)	0.048 (2)	0.064 (3)	0.019 (2)
C39	0.086 (3)	0.133 (4)	0.133 (4)	0.062 (3)	0.037 (3)	0.031 (3)
C40	0.097 (3)	0.105 (3)	0.096 (3)	0.062 (2)	0.037 (2)	0.037 (2)
C41	0.0597 (16)	0.0582 (17)	0.0428 (15)	0.0271 (14)	0.0105 (13)	0.0049 (12)
C42	0.0602 (16)	0.0558 (17)	0.0435 (15)	0.0262 (14)	0.0107 (13)	0.0091 (12)
C43	0.0668 (18)	0.0590 (18)	0.0464 (16)	0.0270 (15)	0.0068 (13)	−0.0002 (13)
C44	0.078 (2)	0.070 (2)	0.064 (2)	0.0184 (18)	0.0174 (17)	−0.0032 (17)
C45	0.111 (3)	0.069 (2)	0.095 (3)	0.000 (2)	0.023 (3)	−0.016 (2)
C46	0.141 (4)	0.085 (3)	0.088 (3)	0.014 (3)	0.017 (3)	−0.037 (3)
C47	0.149 (4)	0.098 (3)	0.075 (3)	0.026 (3)	0.033 (3)	−0.027 (2)
C48	0.106 (3)	0.079 (2)	0.055 (2)	0.028 (2)	0.0250 (19)	−0.0085 (17)
C49	0.0646 (18)	0.0595 (18)	0.0517 (17)	0.0221 (15)	0.0173 (14)	0.0106 (14)
C50	0.097 (2)	0.083 (2)	0.059 (2)	0.047 (2)	0.0255 (18)	0.0180 (17)
C51	0.105 (3)	0.095 (3)	0.074 (2)	0.054 (2)	0.035 (2)	0.012 (2)
C52	0.095 (3)	0.095 (3)	0.060 (2)	0.034 (2)	0.0327 (19)	0.0102 (19)
C53	0.101 (3)	0.104 (3)	0.053 (2)	0.040 (2)	0.0191 (19)	0.0177 (19)
C54	0.092 (2)	0.085 (2)	0.060 (2)	0.045 (2)	0.0193 (18)	0.0199 (18)
C55	0.076 (2)	0.0565 (18)	0.0569 (18)	0.0241 (16)	0.0196 (15)	0.0118 (14)
C56	0.080 (3)	0.075 (2)	0.098 (3)	0.025 (2)	0.023 (2)	0.008 (2)
C57	0.084 (3)	0.085 (3)	0.137 (4)	0.006 (2)	0.018 (3)	−0.003 (3)
C58	0.137 (4)	0.062 (3)	0.133 (4)	0.016 (3)	0.038 (4)	−0.001 (3)
C59	0.134 (4)	0.066 (3)	0.130 (4)	0.045 (3)	0.041 (3)	0.010 (2)
C60	0.094 (3)	0.063 (2)	0.102 (3)	0.037 (2)	0.027 (2)	0.016 (2)
N1	0.0537 (13)	0.0633 (15)	0.0472 (13)	0.0275 (12)	0.0045 (11)	−0.0044 (11)
N2	0.0539 (13)	0.0634 (15)	0.0448 (13)	0.0280 (12)	0.0050 (10)	−0.0022 (11)
N3	0.0752 (16)	0.0557 (15)	0.0592 (15)	0.0277 (13)	0.0261 (13)	0.0055 (12)
N4	0.0788 (17)	0.0516 (14)	0.0708 (17)	0.0309 (13)	0.0330 (14)	0.0078 (12)
N5	0.0651 (15)	0.0552 (14)	0.0459 (13)	0.0215 (12)	0.0137 (11)	0.0044 (11)
N6	0.0744 (16)	0.0534 (14)	0.0524 (14)	0.0233 (13)	0.0181 (12)	0.0092 (11)
O1	0.0645 (12)	0.0599 (12)	0.0493 (11)	0.0280 (10)	0.0051 (9)	−0.0025 (9)
O2	0.0790 (14)	0.0606 (13)	0.0504 (11)	0.0301 (11)	0.0250 (10)	0.0049 (9)
O3	0.0648 (12)	0.0574 (12)	0.0442 (11)	0.0202 (10)	0.0165 (9)	0.0007 (9)
S1	0.0745 (5)	0.0677 (5)	0.0434 (4)	0.0429 (4)	0.0027 (3)	−0.0004 (3)
S2	0.0676 (5)	0.0544 (4)	0.0583 (4)	0.0291 (4)	0.0179 (4)	0.0036 (3)
S3	0.0691 (5)	0.0532 (4)	0.0457 (4)	0.0218 (4)	0.0080 (3)	0.0026 (3)
Co1	0.0635 (3)	0.0525 (3)	0.0396 (2)	0.02712 (19)	0.01122 (17)	0.00224 (16)

### Geometric parameters (Å, °)

**Table:**

C1—O1	1.254 (3)	C32—C33	1.351 (7)
C1—N1	1.331 (4)	C32—H32	0.93
C1—C3	1.489 (4)	C33—C34	1.397 (5)
C2—N1	1.325 (4)	C33—H33	0.93
C2—N2	1.362 (3)	C34—H34	0.93
C2—S1	1.710 (3)	C35—C40	1.360 (5)
C3—C4	1.377 (4)	C35—C36	1.381 (5)
C3—C8	1.388 (4)	C35—N4	1.447 (4)
C4—C5	1.385 (5)	C36—C37	1.382 (5)
C4—H4	0.93	C36—H36	0.93
C5—C6	1.351 (7)	C37—C38	1.358 (6)
C5—H5	0.93	C37—H37	0.93
C6—C7	1.363 (6)	C38—C39	1.357 (7)
C6—H6	0.93	C38—H38	0.93
C7—C8	1.378 (5)	C39—C40	1.387 (6)
C7—H7	0.93	C39—H39	0.93
C8—H8	0.93	C40—H40	0.93
C9—C10	1.351 (5)	C41—O3	1.264 (3)
C9—C14	1.368 (4)	C41—N5	1.330 (4)
C9—N2	1.444 (4)	C41—C43	1.491 (4)
C10—C11	1.395 (6)	C42—N5	1.331 (4)
C10—H10	0.93	C42—N6	1.362 (4)
C11—C12	1.370 (7)	C42—S3	1.718 (3)
C11—H11	0.93	C43—C44	1.376 (5)
C12—C13	1.343 (7)	C43—C48	1.387 (4)
C12—H12	0.93	C44—C45	1.379 (5)
C13—C14	1.378 (5)	C44—H44	0.93
C13—H13	0.93	C45—C46	1.363 (6)
C14—H14	0.93	C45—H45	0.93
C15—C20	1.359 (4)	C46—C47	1.361 (6)
C15—C16	1.373 (4)	C46—H46	0.93
C15—N2	1.442 (4)	C47—C48	1.369 (5)
C16—C17	1.378 (5)	C47—H47	0.93
C16—H16	0.93	C48—H48	0.93
C17—C18	1.345 (5)	C49—C50	1.368 (5)
C17—H17	0.93	C49—C54	1.370 (5)
C18—C19	1.367 (6)	C49—N6	1.446 (4)
C18—H18	0.93	C50—C51	1.388 (5)
C19—C20	1.380 (5)	C50—H50	0.93
C19—H19	0.93	C51—C52	1.374 (6)
C20—H20	0.93	C51—H51	0.93
C21—O2	1.256 (4)	C52—C53	1.370 (5)
C21—N3	1.339 (4)	C52—H52	0.93
C21—C23	1.490 (4)	C53—C54	1.388 (5)
C22—N3	1.331 (4)	C53—H53	0.93
C22—N4	1.357 (4)	C54—H54	0.93
C22—S2	1.723 (3)	C55—C56	1.369 (5)
C23—C28	1.362 (5)	C55—C60	1.373 (5)
C23—C24	1.378 (4)	C55—N6	1.439 (4)
C24—C25	1.364 (5)	C56—C57	1.395 (6)
C24—H24	0.93	C56—H56	0.93
C25—C26	1.340 (7)	C57—C58	1.372 (7)
C25—H25	0.93	C57—H57	0.93
C26—C27	1.384 (7)	C58—C59	1.342 (7)
C26—H26	0.93	C58—H58	0.93
C27—C28	1.375 (6)	C59—C60	1.381 (6)
C27—H27	0.93	C59—H59	0.93
C28—H28	0.93	C60—H60	0.93
C29—C34	1.359 (5)	O1—Co1	1.920 (2)
C29—C30	1.368 (6)	O2—Co1	1.923 (2)
C29—N4	1.450 (4)	O3—Co1	1.934 (2)
C30—C31	1.377 (6)	S1—Co1	2.2153 (9)
C30—H30	0.93	S2—Co1	2.2169 (11)
C31—C32	1.335 (7)	S3—Co1	2.1985 (10)
C31—H31	0.93		
			
O1—C1—N1	129.8 (3)	C37—C36—H36	120.5
O1—C1—C3	116.1 (2)	C38—C37—C36	121.2 (4)
N1—C1—C3	114.0 (2)	C38—C37—H37	119.4
N1—C2—N2	113.0 (2)	C36—C37—H37	119.4
N1—C2—S1	129.9 (2)	C39—C38—C37	119.5 (4)
N2—C2—S1	117.0 (2)	C39—C38—H38	120.3
C4—C3—C8	118.7 (3)	C37—C38—H38	120.3
C4—C3—C1	120.9 (3)	C38—C39—C40	120.5 (5)
C8—C3—C1	120.3 (3)	C38—C39—H39	119.8
C3—C4—C5	120.3 (4)	C40—C39—H39	119.8
C3—C4—H4	119.9	C35—C40—C39	120.0 (4)
C5—C4—H4	119.9	C35—C40—H40	120
C6—C5—C4	120.1 (4)	C39—C40—H40	120
C6—C5—H5	120	O3—C41—N5	129.1 (3)
C4—C5—H5	120	O3—C41—C43	115.9 (3)
C5—C6—C7	120.8 (4)	N5—C41—C43	115.0 (2)
C5—C6—H6	119.6	N5—C42—N6	114.2 (2)
C7—C6—H6	119.6	N5—C42—S3	129.7 (2)
C6—C7—C8	120.0 (4)	N6—C42—S3	116.1 (2)
C6—C7—H7	120	C44—C43—C48	118.8 (3)
C8—C7—H7	120	C44—C43—C41	120.3 (3)
C7—C8—C3	120.2 (4)	C48—C43—C41	120.9 (3)
C7—C8—H8	119.9	C43—C44—C45	120.6 (3)
C3—C8—H8	119.9	C43—C44—H44	119.7
C10—C9—C14	120.0 (3)	C45—C44—H44	119.7
C10—C9—N2	119.5 (3)	C46—C45—C44	119.6 (4)
C14—C9—N2	120.5 (3)	C46—C45—H45	120.2
C9—C10—C11	119.4 (4)	C44—C45—H45	120.2
C9—C10—H10	120.3	C47—C46—C45	120.6 (4)
C11—C10—H10	120.3	C47—C46—H46	119.7
C12—C11—C10	119.9 (5)	C45—C46—H46	119.7
C12—C11—H11	120.1	C46—C47—C48	120.2 (4)
C10—C11—H11	120.1	C46—C47—H47	119.9
C13—C12—C11	120.3 (4)	C48—C47—H47	119.9
C13—C12—H12	119.9	C47—C48—C43	120.2 (4)
C11—C12—H12	119.9	C47—C48—H48	119.9
C12—C13—C14	120.0 (4)	C43—C48—H48	119.9
C12—C13—H13	120	C50—C49—C54	121.0 (3)
C14—C13—H13	120	C50—C49—N6	119.3 (3)
C9—C14—C13	120.4 (4)	C54—C49—N6	119.6 (3)
C9—C14—H14	119.8	C49—C50—C51	119.6 (3)
C13—C14—H14	119.8	C49—C50—H50	120.2
C20—C15—C16	119.2 (3)	C51—C50—H50	120.2
C20—C15—N2	120.4 (3)	C52—C51—C50	119.8 (4)
C16—C15—N2	120.4 (3)	C52—C51—H51	120.1
C15—C16—C17	119.9 (3)	C50—C51—H51	120.1
C15—C16—H16	120	C53—C52—C51	120.1 (3)
C17—C16—H16	120	C53—C52—H52	120
C18—C17—C16	121.1 (4)	C51—C52—H52	120
C18—C17—H17	119.5	C52—C53—C54	120.3 (4)
C16—C17—H17	119.5	C52—C53—H53	119.8
C17—C18—C19	119.1 (4)	C54—C53—H53	119.8
C17—C18—H18	120.4	C49—C54—C53	119.2 (4)
C19—C18—H18	120.4	C49—C54—H54	120.4
C18—C19—C20	120.5 (4)	C53—C54—H54	120.4
C18—C19—H19	119.8	C56—C55—C60	119.6 (3)
C20—C19—H19	119.8	C56—C55—N6	119.4 (3)
C15—C20—C19	120.2 (3)	C60—C55—N6	120.9 (3)
C15—C20—H20	119.9	C55—C56—C57	119.3 (4)
C19—C20—H20	119.9	C55—C56—H56	120.3
O2—C21—N3	129.2 (3)	C57—C56—H56	120.3
O2—C21—C23	115.1 (3)	C58—C57—C56	120.1 (4)
N3—C21—C23	115.7 (3)	C58—C57—H57	120
N3—C22—N4	114.2 (3)	C56—C57—H57	120
N3—C22—S2	130.4 (2)	C59—C58—C57	120.2 (4)
N4—C22—S2	115.3 (2)	C59—C58—H58	119.9
C28—C23—C24	118.7 (3)	C57—C58—H58	119.9
C28—C23—C21	121.3 (3)	C58—C59—C60	120.3 (4)
C24—C23—C21	120.0 (3)	C58—C59—H59	119.8
C25—C24—C23	121.5 (4)	C60—C59—H59	119.8
C25—C24—H24	119.2	C55—C60—C59	120.4 (4)
C23—C24—H24	119.2	C55—C60—H60	119.8
C26—C25—C24	119.4 (4)	C59—C60—H60	119.8
C26—C25—H25	120.3	C2—N1—C1	126.2 (2)
C24—C25—H25	120.3	C2—N2—C15	123.3 (2)
C25—C26—C27	120.6 (4)	C2—N2—C9	119.4 (2)
C25—C26—H26	119.7	C15—N2—C9	117.3 (2)
C27—C26—H26	119.7	C22—N3—C21	123.7 (3)
C28—C27—C26	119.6 (5)	C22—N4—C35	122.7 (3)
C28—C27—H27	120.2	C22—N4—C29	121.3 (2)
C26—C27—H27	120.2	C35—N4—C29	115.9 (2)
C23—C28—C27	120.1 (4)	C41—N5—C42	124.2 (2)
C23—C28—H28	120	C42—N6—C55	121.9 (2)
C27—C28—H28	120	C42—N6—C49	121.0 (2)
C34—C29—C30	119.9 (4)	C55—N6—C49	116.9 (2)
C34—C29—N4	119.5 (3)	C1—O1—Co1	130.12 (18)
C30—C29—N4	120.5 (4)	C21—O2—Co1	129.07 (18)
C29—C30—C31	119.5 (5)	C41—O3—Co1	126.90 (19)
C29—C30—H30	120.2	C2—S1—Co1	106.23 (10)
C31—C30—H30	120.2	C22—S2—Co1	103.40 (11)
C32—C31—C30	121.0 (5)	C42—S3—Co1	106.22 (10)
C32—C31—H31	119.5	O1—Co1—O2	85.41 (9)
C30—C31—H31	119.5	O1—Co1—O3	87.12 (9)
C31—C32—C33	120.1 (4)	O2—Co1—O3	85.99 (9)
C31—C32—H32	119.9	O1—Co1—S3	89.85 (7)
C33—C32—H32	119.9	O2—Co1—S3	175.21 (7)
C32—C33—C34	120.2 (4)	O3—Co1—S3	93.07 (7)
C32—C33—H33	119.9	O1—Co1—S1	95.85 (7)
C34—C33—H33	119.9	O2—Co1—S1	92.24 (7)
C29—C34—C33	119.2 (4)	O3—Co1—S1	176.42 (7)
C29—C34—H34	120.4	S3—Co1—S1	88.94 (4)
C33—C34—H34	120.4	O1—Co1—S2	177.27 (7)
C40—C35—C36	119.8 (3)	O2—Co1—S2	92.85 (7)
C40—C35—N4	120.8 (3)	O3—Co1—S2	90.66 (7)
C36—C35—N4	119.4 (3)	S3—Co1—S2	91.86 (4)
C35—C36—C37	119.0 (4)	S1—Co1—S2	86.31 (4)
C35—C36—H36	120.5		
			
O1—C1—C3—C4	−4.2 (4)	N2—C2—N1—C1	174.2 (3)
N1—C1—C3—C4	175.6 (3)	S1—C2—N1—C1	−4.0 (4)
O1—C1—C3—C8	177.5 (3)	O1—C1—N1—C2	9.7 (5)
N1—C1—C3—C8	−2.7 (4)	C3—C1—N1—C2	−170.1 (3)
C8—C3—C4—C5	0.6 (5)	N1—C2—N2—C15	178.7 (2)
C1—C3—C4—C5	−177.7 (3)	S1—C2—N2—C15	−2.9 (4)
C3—C4—C5—C6	−0.4 (6)	N1—C2—N2—C9	−3.1 (4)
C4—C5—C6—C7	0.0 (7)	S1—C2—N2—C9	175.3 (2)
C5—C6—C7—C8	0.2 (8)	C20—C15—N2—C2	−79.4 (4)
C6—C7—C8—C3	0.1 (7)	C16—C15—N2—C2	103.1 (4)
C4—C3—C8—C7	−0.5 (5)	C20—C15—N2—C9	102.3 (4)
C1—C3—C8—C7	177.8 (3)	C16—C15—N2—C9	−75.2 (4)
C14—C9—C10—C11	−0.1 (7)	C10—C9—N2—C2	100.3 (4)
N2—C9—C10—C11	179.8 (4)	C14—C9—N2—C2	−79.9 (4)
C9—C10—C11—C12	0.7 (9)	C10—C9—N2—C15	−81.4 (4)
C10—C11—C12—C13	−0.3 (9)	C14—C9—N2—C15	98.5 (3)
C11—C12—C13—C14	−0.7 (8)	N4—C22—N3—C21	−178.5 (3)
C10—C9—C14—C13	−0.9 (5)	S2—C22—N3—C21	−0.8 (5)
N2—C9—C14—C13	179.2 (3)	O2—C21—N3—C22	−9.2 (5)
C12—C13—C14—C9	1.3 (6)	C23—C21—N3—C22	172.3 (3)
C20—C15—C16—C17	0.3 (6)	N3—C22—N4—C35	−174.9 (3)
N2—C15—C16—C17	177.9 (3)	S2—C22—N4—C35	7.1 (4)
C15—C16—C17—C18	−0.7 (7)	N3—C22—N4—C29	2.1 (4)
C16—C17—C18—C19	0.7 (8)	S2—C22—N4—C29	−176.0 (3)
C17—C18—C19—C20	−0.3 (9)	C40—C35—N4—C22	−102.6 (4)
C16—C15—C20—C19	0.0 (6)	C36—C35—N4—C22	79.8 (4)
N2—C15—C20—C19	−177.5 (4)	C40—C35—N4—C29	80.2 (4)
C18—C19—C20—C15	0.0 (8)	C36—C35—N4—C29	−97.3 (4)
O2—C21—C23—C28	−176.1 (4)	C34—C29—N4—C22	−93.6 (4)
N3—C21—C23—C28	2.6 (5)	C30—C29—N4—C22	87.4 (5)
O2—C21—C23—C24	2.7 (5)	C34—C29—N4—C35	83.6 (4)
N3—C21—C23—C24	−178.6 (3)	C30—C29—N4—C35	−95.4 (4)
C28—C23—C24—C25	−1.3 (6)	O3—C41—N5—C42	3.5 (5)
C21—C23—C24—C25	179.9 (4)	C43—C41—N5—C42	−174.3 (3)
C23—C24—C25—C26	2.1 (8)	N6—C42—N5—C41	167.0 (3)
C24—C25—C26—C27	−1.6 (11)	S3—C42—N5—C41	−13.9 (5)
C25—C26—C27—C28	0.3 (12)	N5—C42—N6—C55	−175.0 (3)
C24—C23—C28—C27	−0.1 (8)	S3—C42—N6—C55	5.8 (4)
C21—C23—C28—C27	178.7 (5)	N5—C42—N6—C49	10.1 (4)
C26—C27—C28—C23	0.6 (11)	S3—C42—N6—C49	−169.0 (2)
C34—C29—C30—C31	−0.9 (7)	C56—C55—N6—C42	−110.8 (4)
N4—C29—C30—C31	178.2 (4)	C60—C55—N6—C42	69.3 (4)
C29—C30—C31—C32	−0.9 (9)	C56—C55—N6—C49	64.3 (4)
C30—C31—C32—C33	2.4 (9)	C60—C55—N6—C49	−115.7 (4)
C31—C32—C33—C34	−2.3 (8)	C50—C49—N6—C42	63.7 (4)
C30—C29—C34—C33	1.0 (6)	C54—C49—N6—C42	−118.7 (4)
N4—C29—C34—C33	−178.0 (3)	C50—C49—N6—C55	−111.4 (4)
C32—C33—C34—C29	0.5 (7)	C54—C49—N6—C55	66.2 (4)
C40—C35—C36—C37	0.4 (5)	N1—C1—O1—Co1	1.0 (4)
N4—C35—C36—C37	178.0 (3)	C3—C1—O1—Co1	−179.22 (17)
C35—C36—C37—C38	−1.1 (6)	N3—C21—O2—Co1	−15.1 (5)
C36—C37—C38—C39	1.2 (7)	C23—C21—O2—Co1	163.5 (2)
C37—C38—C39—C40	−0.6 (7)	N5—C41—O3—Co1	29.3 (4)
C36—C35—C40—C39	0.1 (6)	C43—C41—O3—Co1	−152.9 (2)
N4—C35—C40—C39	−177.4 (4)	N1—C2—S1—Co1	−7.9 (3)
C38—C39—C40—C35	−0.1 (7)	N2—C2—S1—Co1	174.04 (18)
O3—C41—C43—C44	−2.2 (4)	N3—C22—S2—Co1	24.0 (3)
N5—C41—C43—C44	175.9 (3)	N4—C22—S2—Co1	−158.4 (2)
O3—C41—C43—C48	177.8 (3)	N5—C42—S3—Co1	−4.7 (3)
N5—C41—C43—C48	−4.1 (5)	N6—C42—S3—Co1	174.3 (2)
C48—C43—C44—C45	1.0 (6)	C1—O1—Co1—O2	−102.9 (2)
C41—C43—C44—C45	−179.0 (4)	C1—O1—Co1—O3	170.9 (2)
C43—C44—C45—C46	−0.5 (7)	C1—O1—Co1—S3	77.8 (2)
C44—C45—C46—C47	−1.5 (9)	C1—O1—Co1—S1	−11.1 (2)
C45—C46—C47—C48	2.9 (9)	C21—O2—Co1—O1	−144.6 (3)
C46—C47—C48—C43	−2.4 (8)	C21—O2—Co1—O3	−57.2 (3)
C44—C43—C48—C47	0.4 (6)	C21—O2—Co1—S1	119.7 (3)
C41—C43—C48—C47	−179.6 (4)	C21—O2—Co1—S2	33.3 (3)
C54—C49—C50—C51	−0.6 (6)	C41—O3—Co1—O1	−127.1 (2)
N6—C49—C50—C51	177.1 (3)	C41—O3—Co1—O2	147.3 (2)
C49—C50—C51—C52	0.3 (6)	C41—O3—Co1—S3	−37.4 (2)
C50—C51—C52—C53	0.3 (6)	C41—O3—Co1—S2	54.5 (2)
C51—C52—C53—C54	−0.6 (6)	C42—S3—Co1—O1	109.02 (12)
C50—C49—C54—C53	0.3 (6)	C42—S3—Co1—O3	21.91 (12)
N6—C49—C54—C53	−177.4 (3)	C42—S3—Co1—S1	−155.12 (11)
C52—C53—C54—C49	0.3 (6)	C42—S3—Co1—S2	−68.85 (11)
C60—C55—C56—C57	−1.1 (6)	C2—S1—Co1—O1	11.72 (12)
N6—C55—C56—C57	179.0 (4)	C2—S1—Co1—O2	97.33 (12)
C55—C56—C57—C58	0.9 (7)	C2—S1—Co1—S3	−78.02 (10)
C56—C57—C58—C59	−0.4 (9)	C2—S1—Co1—S2	−169.95 (10)
C57—C58—C59—C60	0.2 (9)	C22—S2—Co1—O2	−30.16 (12)
C56—C55—C60—C59	0.9 (6)	C22—S2—Co1—O3	55.86 (12)
N6—C55—C60—C59	−179.2 (4)	C22—S2—Co1—S3	148.96 (11)
C58—C59—C60—C55	−0.4 (7)	C22—S2—Co1—S1	−122.23 (11)
